# The Potential Role of Regenerative Medicine on the Future Management of Hypoplastic Left Heart Syndrome

**DOI:** 10.3390/jcdd9040107

**Published:** 2022-04-02

**Authors:** John M. Kelly, Cole Anderson, Christopher K. Breuer

**Affiliations:** 1 Center for Regenerative Medicine, Abigail Wexner Research Institute at Nationwide Children’s Hospital, Columbus, OH 43205, USA; john.kelly@nationwidechildrens.org; 2 Department of Pediatrics, The Ohio State University College of Medicine, Columbus, OH 43210, USA; 3 The Heart Center, Nationwide Children’s Hospital, Columbus, OH 43205, USA; 4 Biomedical Engineering Graduate Program, The Ohio State University, Columbus, OH 43210, USA; cole.anderson@nationwidechildrens.org; 5 Department of Surgery, The Ohio State University Wexner Medical Center, Columbus, OH 43210, USA; 6 Department of Surgery, Nationwide Children’s Hospital, Columbus, OH 43205, USA

**Keywords:** regenerative medicine, hypoplastic left heart syndrome

## Abstract

The development and translation of regenerative medicine approaches for the treatment of hypoplastic left heart syndrome (HLHS) provides a promising alternative to the current standard of care. We review the strategies that have been pursued to date and those that hold the greatest promise in moving forward. Significant challenges remain. Continued scientific advances and technological breakthroughs will be required if we are to translate this technology to the clinic and move from palliative to curative treatment.

## 1. Introduction

Hypoplastic Left Heart Syndrome (HLHS) is a critical congenital cardiac anomaly defined by a hypoplastic left ventricle, mitral and aortic valve stenosis/atresia, and a diminutive ascending aorta [1]. It is generally considered fatal without treatment. The typical treatment approach requires three open-heart procedures before the age of 2–4 years of life, culminating in the Fontan operation [2]. After completion of the Fontan circulation, the functional right ventricle pumps blood to the systemic circulation while the systemic venous return is rerouted to deliver deoxygenated blood passively to the pulmonary circulation [3]. While lifesaving, the Fontan operation is a palliative, noncurative operation associated with increased morbidity and mortality and decreased quality of life [4]. Heart failure symptoms can be precipitated by classic ventricular pump failure or occur in the setting of normal ventricular function in what is classified as Fontan circulatory failure. These adverse changes are largely believed to be driven by the abnormal Fontan hemodynamics characterized by non-pulsatile pulmonary blood flow, chronic venous hypertension, and preload deprivation [3]. Beyond heart transplantation, there are generally no effective medical or surgical therapies for treating Fontan failure and few for slowing its progression [4]

Regenerative medicine is a multidisciplinary endeavor that attempts to leverage our cells’ innate capacity to multiply and self-organize into functional units or neotissues. The resulting neotissues can be used to surgically repair or replace tissues that are either diseased, damaged, or congenitally absent. Since its inception, regenerative medicine has held promise for advancing the field of congenital heart surgery under the premise that regenerative medicine techniques could be used to create autologous cardiovascular neotissues, which, in turn, could be used to repair or replace malformed or congenitally absent tissues such as those associated with HLHS [5]. 

## 2. Regenerative Medicine Approaches to Date Applicable to Patients with HLHS

### 2.1. Generation of Vascular Conduits and Valves

While some promising early preclinical studies highlighted the potential to use tissue-engineering methods to create cardiovascular patches, vascular conduits, and replacement heart valves, the development and translation of this technology to the clinic have been slow [6]. The first in human studies and early clinical trials evaluating the use of tissue-engineered vascular patches and conduits in congenital heart surgery were initiated in 2000 [7]. The results of these pioneering studies were quite promising and demonstrated the feasibility of using a regenerative medicine strategy in humans. Unfortunately, the early promising results and associated enthusiasm for tissue engineering waned when one of the early clinical trials evaluating the use of tissue-engineered heart valves in humans was put on hold when several patients died unexpectantly from catastrophic valve failure necessitating the remaining patients in the study have their tissue-engineered heart valves removed prophylactically [8]. Analysis of the tissue-engineered heart valves revealed that the scaffolds created from recellularized xenograft heart valves had not been adequately decellularized, resulting in acute rejection. These results highlighted the need for regulatory supervision. While the need for regulatory oversite was clear and critical for improving the safety of these early clinical studies, it delayed the translation of tissue-engineering technology to the clinic as the regulatory agencies needed time to develop new methods and strategies for evaluating and monitoring regenerative medicine products.

More recently, results of clinical trials evaluating the use of autologous tissue-engineered constructs developed using either cell-seeded or unseeded scaffolds, which provide sites for cell attachment and space for neotissue formation while also serving as a template to direct neotissue formation, have moved beyond being the first in human studies, and into late-stage clinical trials in Europe and the United States [9,10]. Furthermore, clinical and preclinical studies have demonstrated these tissue-engineering methods can be used to create autologous living structures that mimic the structure and functions of their native counterparts exhibiting a functional endothelium, which lowers the risk of thromboembolic complications after scaffold degradation is complete [11]. The biomechanics and vasoreactivity of these constructs also mimic those of their native counterparts, which has important implications for their hemodynamic performance and long-term function [12].

Finally, the creation of living neotissues with growth capacity enables the avoidance of somatic overgrowth, a significant concern in all major congenital heart operations, especially in those performed in the newborn period [12]. Recent advances in our mechanistic understanding of the cellular and molecular processes underlying neotissue formation, coupled with advances in computational modeling, could accelerate the development and translation of these technologies [13]. Despite these advances, including clinical trials that evaluate tissue-engineered constructs in patients with HLHS [14], even if these are successful, these advances would only incrementally improve our ability to treat HLHS. They have been applied to develop vascular conduits and valves but do not replace or augment the hypoplastic left ventricular tissue and thus, still only provide a palliative and noncurative treatment strategy.

### 2.2. Myocardial Preservation

In vivo cell-based therapies to regenerate damaged myocardial tissue in response to myocardial ischemia have generated significant interest in preclinical and clinical studies [15]. Variable results with modest to no improvement in myocardial scar burden and ventricular function have been demonstrated [16]. Cell source, delivery method, and timing of delivery relative to the ischemic insult are of important consideration. However, any benefit is not mediated by stem cell engraftment and differentiation to cardiomyocytes but rather by paracrine pathways modulating the adverse remodeling associated with myocardial infarction [17]. Similarly, cell-based therapies have been applied to patients with hypoplastic left heart syndrome [18].

The general treatment paradigm promotes favorable right ventricular remodeling, which structurally and functionally did not evolve to support the systemic circulation and must function within the foreign hemodynamic environment of Fontan palliation and multiple prior exposures cardiopulmonary bypass and cardioplegia through staged surgical palliation. Similar to the adult experience, only modest improvements in ventricular function and size have been demonstrated [19,20]. Future studies further defining the ideal cell source, treatment timing/frequency, and ideal method of delivery are required.

## 3. Development of a Curative Treatment Strategy for HLHS

### 3.1. Fetal Strategies

One potential method for the treatment and prevention of HLHS includes transcatheter fetal intervention with the delivery of current scaffold-based tissue-engineering techniques described above to leverage the unique features of the fetal environment. Early clinical fetal valvuloplasty studies demonstrated that approximately one-third of patients with severe midgestational aortic stenosis that are at risk for progression to hypoplastic left heart syndrome (HLHS) could be converted to biventricular anatomy by performing fetal aortic valvuloplasty, thus restoring flow through the left ventricle and preventing the left ventricular hypoplasia [21]. Building on this success, recent preclinical pilot studies have demonstrated the ability to replace fetal heart valves by utilizing tissue-engineered replacement valves by way of catheter-based techniques, all during the same points in gestation as the clinical fetal valvuloplasty was performed [22]. While at a very nascent stage, this pathway holds promise for developing a regenerative medicine strategy of creating autologous ventricular tissue without constructing this neotissue from its cellular components. Significant hurdles remain, including identifying the subpopulations of patients with HLHS who would be amenable to this treatment. Two-thirds of the patients in the valvuloplasty trial failed to respond adequately. This indicates possible underlying cardiomyocyte abnormalities which persist despite the reinstitution of more normal hemodynamics [23]. Furthermore, the determination of the optimal timing for intervention will need to be defined. Significant technological advancements will be required, including improved imaging, better delivery systems, customized tissue-engineered scaffolding, and robotics to consider potentially developing and translating this approach to the clinic.

### 3.2. Restoration of Normal Cardiac Function

The holy grail of regenerative medicine strategies applied to patients born with HLHS or patients with HLHS and Fontan failure includes the creation of autologous pulsatile cardiac tissue to supplement the diminutive left ventricle and autologous replacement donor hearts.

### 3.3. Whole Organ Transplantation

Early preclinical studies using decellularization-recellularization tissue-engineering methods demonstrated the concept of using regenerative medicine techniques to create whole organs, including the heart [24]. When coupled with the development of iPS cell technology, this methodology was met with great enthusiasm. However, like the scaffold-based tissue-engineering approaches, the early process of developing and translating this technology to the clinic has also proven to be more difficult than initially imagined [25]. Recent advances in xenotransplantation and genetic engineering hold promise to produce replacement donor hearts with the first successful pig-to-human transplant recently reported [26]. Important questions remain unanswered related to what genetic modifications are required to reduce the risk of rejection and promote an appropriate growth response to endogenous hormones. 

### 3.4. Regeneration of Cardiomyocytes

While the promise of iPSC methods for creating autologous cells for regenerative medicine applications is great, so too are its complexities. Fundamental biological questions such as which cell types are required to make functional pulsatile cardiac tissues of adequate purity that will stably integrate into the host remain to be answered [27]. Successful electromechanical coupling, vascularization to produce viable tissue at the scale of the human heart, and appropriately mediating the immune response all need to be addressed [28]. 

Developing strategies to directly connect or enable inosculation into the host vasculature are also areas of focus and advancement. The application of bioprinting coupled with advancements in bioreactors have demonstrated promise in early preclinical studies for assembling, maturing, and preserving these tissue-engineered constructs before implantation but is at an early stage of development [29,30]. Significant manufacturing challenges remain to scale and deliver this technology to the bedside. From a regulatory standpoint, the possibility of malignant dedifferentiation must be addressed before translation, while the introduction of new, unanticipated complications must be considered. Results of late-stage preclinical studies evaluating the use of iPS-derived cardiomyocyte cell therapy for heart failure have revealed new hurdles, such as the development of arrhythmias highlighting the enormous complexity and amount of basic mechanistic work required to advance the field and enable continued progress using this strategy [31]. Furthermore, the application of patient-specific iPSCs to produce functional cardiomyocytes may be limited by poor iPSC yield and impaired cardiac function secondary to the genetic underpinnings of the disease [32]. Important ethical and regulatory issues specific to the pediatric population will need to be addressed as we consider this potential treatment strategy as an alternative to the current standard of care for patients with HLHS [33].

## 4. Conclusions

Continued scientific advances and technological breakthroughs will be required if we are to move beyond the palliative and towards curative treatments for critical cardiac anomalies. The development and translation of regenerative medicine approaches for the treatment of HLHS provides a promising potential approach towards achieving this goal, but significant challenges and enormous amounts of research will be required if we pursue this approach. We recently celebrated the 50th anniversary of the first successful heart transplant, an amazing feat built upon a half-century of research [34]. Where will we be in 50 years? Do we possess the drive, innovation, and collaboration to successfully develop and translate a regenerative medicine strategy for the treatment of HLHS? Only time will tell. 

## Data Availability

No datasets relevant to this manuscript.
